# Fixed-dose combination orally disintegrating tablets to treat cardiovascular disease: formulation, in vitro characterization and physiologically based pharmacokinetic modeling to assess bioavailability

**DOI:** 10.2147/DDDT.S126035

**Published:** 2017-03-16

**Authors:** Thomas J Dennison, Julian C Smith, Raj K Badhan, Afzal R Mohammed

**Affiliations:** 1 Aston School of Pharmacy, Aston University, Birmingham; 2 Viridian Pharma Ltd, Newport, UK

**Keywords:** orally disintegrating tablet, fixed-dose combination, cardiovascular disease, physiologically based pharmacokinetic modeling, bioavailability, bioequivalence

## Abstract

Cardiovascular disease (CVD) is the leading cause of death among men and women worldwide. In CVD, hypertension and dyslipidemia commonly coexist and are managed through coadministration of amlodipine and atorvastatin, respectively. The case for fixed-dose combination (FDC) oral dosage forms and orally disintegrating tablet (ODT) technology to enhance outcomes and compliance is strong. This work follows the development and characterization of single and FDC ODTs containing amlodipine and atorvastatin, followed by bioequivalence comparison between these single and FDC formulations, using in vitro dissolution and Caco-2 apparent permeability (P_app_) and in silico physiologically based pharmacokinetic modeling approaches. ODTs containing amlodipine (5 mg) and atorvastatin (10 mg) either alone or in combination rapidly disintegrated (<30 s) while displaying a radial crushing strength in excess of 100 N and friability ≤1%. In vitro dissolution test was performed in fasted and fed-state simulated intestinal fluid (FeSSIF) and analyzed using high-performance liquid chromatography. Dissolution profiles for single and FDC ODTs were compared using US FDA recommended difference (f_1_) and similarity (f_2_) factor testing for bioequivalence. In all cases, there was no difference in active pharmaceutical ingredient dissolution between single or FDC ODTs, with the exception of amlodipine in FeSSIF. Pharmacokinetic clinical trial simulations were conducted using Simcyp (Version 14), incorporating P_app_ and dissolution data. Simulated clinical trials in healthy volunteers showed no difference in bioavailability based on pharmacokinetic parameters between single and combination doses with either active pharmaceutical ingredient. An increase in C_max_ and AUC for atorvastatin in fed subjects was attributed to extended transit along the gut lumen and reduced atorvastatin metabolism due to lower CYP3A4 expression at more distal small intestine absorption sites. The results demonstrated bioequivalence of an FDC ODT for amlodipine and atorvastatin, while highlighting several limitations of f_1_ and f_2_ bioequivalence testing and strengths of mechanistic pharmacokinetic modeling for oral drug absorption.

## Introduction

Cardiovascular disease (CVD) is the leading cause of death worldwide, claiming an estimated 17.3 million lives per year, a death toll that is expected to rise to in excess of 23.6 million by 2030. Deaths from CVD accounted for 30% of global deaths in 2008, more than all forms of cancer combined.^1^

CVD is multifactorial, with risk factors such as hypertension, dyslipidemia, diabetes mellitus, smoking, and obesity frequently coexisting.^2^ One of the most common risk factor combinations is dyslipidemia (elevated levels of low-density lipoprotein [LDL] and triglyceride and low levels of high-density lipoprotein [HDL]) and hypertension.^3^ Studies have demonstrated the link between hypertension and metabolically associated risk factors;^4^ in a retrospective study of US veterans, for example, the prevalence of CVD was commonly double in patients exhibiting both hypertension and dyslipidemia when compared to those with either condition alone.^5^ In the UK, a 2004 analysis of the medical records of over 600,000 patients revealed a 14.7% incidence of subjects with both hypertension and dyslipidemia.^6^

Amlodipine (Biopharmaceutics Classification System [BCS] class I)^7^ is a third-generation dihydropyridine calcium channel blocker, a class of drug that works to lower blood pressure in hypertensive patients through relaxation of vascular smooth muscle and vessel dilation.^8^ It acts by inhibiting “slow” influx of extracellular calcium into cardiac and vascular cells via blockade of voltage-gated L-type calcium channels.^9^,^10^ Amlodipine’s slow onset of action is responsible for a low incidence of reflex tachycardia and other vasodilator side effects when compared to other dihydropyridines, while its slow elimination and resultant long duration of action grants the convenience of a once-daily dosage regime.^11^

Atorvastatin (BCS class II),^12^ a 3-hydroxy-3-methylglutaryl coenzyme A (HMG-CoA) reductase inhibitor, is used extensively in the treatment of dyslipidemia.^13^ HMG-CoA reductase catalyzes the conversion of HMG-CoA to mevalonate. Its inhibition reduces hepatocyte cholesterol levels, leading to upregulation of LDL cholesterol (LDL-C) cell surface receptors and resulting in increased clearance of LDL-C from plasma.^14^,^15^ Atorvastatin reportedly reduces LDL-C in hypercholesterolemic patients by 41%–61%^16^ and reduces total cholesterol and plasma triglycerides alongside a modest increase in HDL holesterol (HDL-C) levels.^17^

Despite the substantial risk of patients suffering from both dyslipidemia and hypertension, successful treatment falls short.^18^ A major reason for this is poor patient compliance, for reasons including cost, treatment regime complexity, extent of concomitant treatment, and side effects.^16^,^19^,^20^ Several clinical studies have examined the efficacy and safety of amlodipine and atorvastatin combination therapy in patients with concurrent hypertension and dyslipidemia. Combination therapy has been shown to achieve blood pressure and LDL goals.^21^,^22^ The RESPOND study, which compared combination therapy with amlodipine or atorvastatin, alone showed no difference in efficacy,^23^ whereas the AVALON study reported an increased efficacy with combination therapy over either drug alone.^24^ Furthermore, when amlodipine and atorvastatin are administered in a fixed-dose combination (FDC), there is no significant difference in bioavailability (based on t_max_, C_max_, and AUC) compared to coadministered matching doses of individual amlodipine and atorvastatin tablets.^25^

An amlodipine and atorvastatin FDC is, therefore, an attractive prospect with the view of improving patient compliance. In addition to demonstrating bioequivalence in vivo, in combination, both amlodipine and atorvastatin allow for once-daily dosing and have no issues with tolerability.^16^ Indeed, an amlodipine and atorvastatin FDC (Caduet^®^; Pfizer, New York, NY, USA) was approved in 2004 as the first FDC to treat two CVD categories.^26^

Orally disintegrating tablets (ODTs) are an appealing solid dosage form that rapidly disintegrates upon contact with saliva, typically within 30 s, eliminating the need for swallowing.^27^ This is pertinent to patients with dysphagia, a difficulty in swallowing, a condition estimated to affect as much as 50% of the population,^28^ while a recent patient survey across 11 general practices reported an incidence of 37.4%.^29^ Dysphagia is particularly prevalent in pediatric and geriatric populations, institutionalized and psychiatric patients, those suffering from nausea and vomiting, and individuals with lack of access to water.^30^,^31^ Other benefits of ODTs include accurate dosing, rapid onset of action, good mouth feel, new business opportunities, and low production costs.^30^

In the application for and approval of generic medicinal formulations, the demonstration of bioequivalence is fundamental. Bioequivalence is based on the assumption that when two medicinal products display equivalent bioavailability, they will have the same therapeutic effect and thus provide comparable in vivo performance, in terms of both efficacy and safety.^32^ A product is deemed bioequivalent when there is no significant difference in the rate and extent to which the active pharmaceutical ingredient (API) becomes available within the systemic circulation, when compared with a reference drug product.^33^ Bioequivalence testing may also be applied in other situations, including the assessment of FDCs.^32^ For immediate release formulations, in vitro dissolution testing can be used to waive bioequivalence requirements, with the US FDA recommending a dissolution profile comparison approach, comprising a difference factor (f_1_) and similarity factor (f_2_).^33^,^34^ For rapidly dissolving medicinal products displaying greater than 85% dissolution within 15 min, comparison testing is not necessary, under the condition that the API falls within BCS class I or III (although class III carries stricter requirements).^32^ The potential for biowaiver extension to BCS class II compounds is an area of much interest.^35^–^37^

Over the past 20 years, pharmacokinetic modeling and simulation have become an established tool to improve efficiency and reduce cost during drug development and ADME (absorption, distribution, metabolism and excretion) assessment. Physiologically based pharmacokinetic (PBPK) modeling describes the tissues and organs in the body as defined compartments, which are assigned physiologically relevant parameters and connected via physiological perfusion rates.^38^ PBPK models are used to estimate the pharmacokinetic profile of a drug at a target tissue or organ by taking into account ADME considerations throughout all compartments.^39^,^40^ As such, PBPK models have become a powerful tool for prediction of oral drug absorption (to the systemic circulation) through integration of common in vitro drug-specific information, such as physicochemical and cell-based permeability data, with systems-based (physiological, anatomical, and biochemical) data.^41^,^42^ PBPK modeling is often exploited for prediction of oral drug absorption, concerning the effect of formulation changes^43^,^44^ or FDCs,^45^ for example, and there is a significant effort to employ PBPK modeling to determine bioequivalence.^46^–^48^

The potential to enhance therapy for patients suffering both dyslipidemia and hypertension with an orally disintegrating FDC for amlodipine and atorvastatin is substantial. Since no change in bioavailability for amlodipine and atorvastatin from FDCs has been reported, it is expected that FDC ODTs, given their immediate disintegration and therefore rapid drug release, should show similar findings. Furthermore, the ability of ODTs to increase patient compliance due to their convenience as a dosage form would likely enhance CVD therapy. In this work, an FDC ODT for amlodipine and atorvastatin was developed and characterized. Single-dose and fixed-dose drug dissolution from ODTs were tested in biorelevant media, while drug permeability across Caco-2 cell monolayers was measured for prediction of in vivo pharmacokinetics and bioequivalence of FDCs compared to single-dose formulations, through PBPK computational modeling.

## Materials and methods

### Materials

Amlodipine besylate (herein referred to as amlodipine) was purchased from Molekula Ltd (Gillingham, UK) and atorvastatin calcium (herein referred to as atorvastatin) from Chemical Point (Oberhaching, Germany). Pearlitol^®^ Flash (mannitolstarch copolymer) was obtained from Roquette Pharma (Les-trem, France), and Avicel PH-102 micro-crystalline cellulose (MCC) and sodium stearyl fumarate (SSF) were purchased from FMC BioPolymer (Philadelphia, PA, USA).

Biorelevant fasted-state simulated intestinal fluid (FaSSIF)/fed-state simulated intestinal fluid (FeSSIF)/fasted state simulated gastric fluid (FaSSGF) Instant Powder was purchased online from biorelevant.com (UK). Sodium hydroxide, sodium chloride, sodium phosphate, and glacial acetic acid for biorelevant media were obtained from Sigma-Aldrich (Gillingham, UK). Acetonitrile (ACN) and methanol (high-performance liquid chromatography [HPLC]-grade) were obtained from Fisher Scientific (Loughborough, UK).

For cell culture media, Dulbecco’s Modified Eagle’s Medium (DMEM) was purchased from Lonza (Wokingham, UK). Fetal bovine serum (FBS), gentamicin (10 mg/mL), Fungizone (amphotericin B 250 μg/mL), Hanks’ balanced salt solution (HBSS), and penicillin/streptomycin (10,000 U/mL) were all purchased from Gibco (Thermo Fischer Scientific). Trypsin-Ethylenediaminetetraacetic acid solution (0.25%) was procured from Sigma-Aldrich (Gillingham, UK). Caco-2 cells were purchased from the European Collection of Authenticated Cell Cultures (ECACC) via Public Health England.

### HPLC

HPLC was performed on an Agilent 1260 series (Agilent Technologies, Santa Clara, CA, USA), comprising a quarternary pump, Infinity variable wavelength detector, and autosampler. Analysis was conducted on a reversed-phase Gemini C18, 150×4.6 mm, 110 Å, 5 μm column (Phenomenex, Macclesfield, UK). Protocols were developed, calibrated, and validated for both amlodipine and atorvastatin alone and in combination.

Separations were achieved using 0.1% (v/v) TFA and ACN at different ratios as the mobile phase. Amlodipine separation was performed with an isocratic mobile phase of TFA:CAN (57.5:42.5 v/v), a flow rate of 1 mL/min, and a wavelength of 360 nm. Atorvastatin separation was achieved using an isocratic mobile phase of TFA:ACN (50:50 v/v), a flow rate of 1.2 mL/min, and a wavelength of 246 nm. Separation of amlodipine and atorvastatin in combination required a mobile phase of TFA:ACN delivered at a gradient (65:35 to 35:65 v/v), with a flow rate of 1.5 mL/min, and a wavelength of 240 nm. An injection volume of 20 μL was used throughout.

HPLC method validation involved assessment of precision through intra-day variation, accuracy by multilevel recovery studies, instrument precision, linearity, and limit of detection and quantification (LOD and LOQ). Stock solutions (1 mg/mL) of each drug were prepared (using ACN and methanol as solvents for amlodipine and atorvastatin, respectively) from which dilutions and subsequently twofold serial dilutions were prepared to form a calibration curve.

### Tablet production

Direct compression of tablets (500 mg) was performed on an Atlas T8 automatic press (SPECAC, Orpington, UK), using a 13 mm round, flat-faced die. Tablets were produced under ambient conditions.

### Friability

Tablet friability was determined on 6 tablets using an F2 friability tester (Sotax, Aesch, Switzerland). Tablets were placed inside a drum and rotated at 25 rpm for a total of 100 revolutions. Dust was removed pre- and posttesting to remove excess powder that would contribute to tablet mass. Friability was calculated and expressed as percent tablet weight loss from initial tablet weight.

### Tablet hardness

A Tablet Hardness Tester TBF1000 (Copley Scientific, Nottingham, UK) was used to measure the radial crushing strength (hardness) of tablets in triplicate.

### Dissolution testing

API dissolution from ODTs in 900 mL biorelevant media was tested in both FaSSIF and FeSSIF at pH 6.5 and 5, respectively, and maintained at 37°C. An ERWEKA DT 600 USP 2 paddle apparatus (Heusenstamm, Germany) was used at a paddle speed of 50 rpm.^27^ A total of 5 mL of sample was taken over 2 h, replacing with 5 mL fresh media to simulate sink conditions. API dissolution was measured using HPLC and corrected for percent dose dissolved.

### Cell culture

Prior to seeding, cells were trypsinized (2.5 mL) from 75 cm^2^ cell culture flasks (Corning Inc., Corning, NY, USA) on which they had been grown (80% confluence), after washing with HBSS. Caco-2 cells (passage 54–58) were seeded onto Transwell (Corning) semi-permeable membrane supports (12 well, 1.12 cm^2^, 0.4 μm pore size) at a density of 8×10^4^ cells/cm^2^. Cells were maintained in DMEM containing l-glutamine (4 mM) and glucose (4.5 mg/mL) supplemented with (v/v) 10% FBS, 1% penicillin/streptomycin, 1% nonessential amino acids, amphotericin B (0.5 μg/mL), and gentamicin (20 μg/mL). Media were changed every 2–3 days and transwells cultured at 37°C, 5% CO_2_ for 21 days, after which transport studies were performed.

### Transepithelial electrical resistance (TEER) measurements

TEER value measurements were performed to monitor monolayer integrity using an EVOM meter (World Precision Instruments, Sarasota, FL, USA). TEER values are expressed using the following equation:

$$\text{TEER}(\Omega \cdot {\text{cm}}^{2})=\text{Resistance}-\text{Blank resistance}\times \text{Membrane surface area}({\text{cm}}^{2})$$

### Caco-2 transport studies

Caco-2 monolayers were used for transport studies between 21 and 24 days post-seeding. Drug absorption through Caco-2 monolayers was measured for amlodipine and atorvastatin alone and in combination in both the apical to basolateral (A–B) and basolateral to apical (B–A) directions (n=3). Transport studies were carried out in DMEM (37°C) containing 10 mM (4-(2-hydroxyethyl)-1-piperazineethanesulfonic acid) (pH 7.4), with 0.5 and 1.5 mL in the A and B compartments, respectively. Samples of 100 μL were removed from the A side and 200 μL from the B side at time points over 2 h, replacing with fresh prewarmed media (37°C) to mimic sink conditions. For mass balance, samples were taken from the donor compartments at t =0 and t =120 min.

Amlodipine was administered at a concentration equivalent to 20 μg/mL (representing a dose of 5 mg in 250 mL) and atorvastatin at a concentration equivalent to 40 μg/mL (representing a dose of 10 mg in 250 mL). Cultures were maintained at 37°C and 5% CO_2_ throughout the experiment. Samples were analyzed by HPLC, and apparent permeability (P_app_) values were calculated using the following equation:

$$P_{\text{app}}=\frac{\left(\text{dQ}/\text{dt}\right)}{\left(C_{0}\times A\right)}$$

where dQ/dt is the mass transfer rate of the compound from the donor to the receiver compartment, C_0_ is the initial concentration in the donor chamber, and A is the monolayer surface area (cm^2^).

### Clinical trials simulation

The population-based clinical trials simulator Simcyp (V14) (Certara, Princeton, NJ, USA) was used to simulate the plasma concentration of atorvastatin and amlodipine from single API and FDC formulations. Default parameter values for creating a North European Caucasian population were selected.^49^

### Compound data

Physicochemical information for each API was collated from the literature and used to develop compound files (Table 1). Simulations were performed using a minimal-PBPK model. Where uncertainty arose regarding the precise value of compound data parameters, parameter estimation was conducted using the Parameter Estimation Module to optimize parameter values. The ADAM (advanced dissolution, absorption and metabolism) model^43^ was assumed for all simulations and the dissolution profile for each formulation (single and FDC) in FaSSIF and FeSSIF was utilized.

### Clinical studies

The optimization and validation of the PBPK model were conducted using clinical study results reported in healthy adult subjects. For atorvastatin, study 1 included a 20 mg tablet dosed to 36 healthy volunteers (18–45 years old),^50^ study 2 included a 20 mg tablet dosed to 24 healthy subjects,^51^ study 3 included an 80 mg capsule dosed to 36 healthy subjects (20–50 years old),^52^ and study 4 included a 10 mg tablet dosed to 50 healthy volunteers.^53^

For amlodipine, study 1 included a 5 mg tablet dosed to 24 healthy subjects,^51^ study 2 included a 5 mg tablet dosed to 28 healthy volunteers (35.48±9.52 years old),^54^ study 3 included a 10 mg tablet dosed to 24 healthy volunteers (21–29 years old),^55^ and study 4 included a 10 mg tablet dosed to 35 subjects (18–46 years old).^56^ In both cases, studies 1 and 2 were used to develop and optimize the compound file before validating with two further clinical studies (studies 3 and 4).

Raw data from published human trial plasma concentration profiles were extracted using WebPlotDigitizer 3.10^57^ and, where necessary, parameter estimation was conducted using the validation clinical datasets.

Predictions of API plasma pharmacokinetic profiles were simulated following the oral administration of a single immediate release solid dosage form of 10 mg (atorvastatin) and 5 mg (amlodipine) dose over a 24 h period.

### Statistical analysis

GraphPad PRISM software version 6.01 (San Diego, CA, USA) was used for data analysis. Ordinary one-way analysis of variance (ANOVA) was used with Tukey’s multiple comparisons test to analyze data for tablet characterization. Unpaired two-tailed *t*-test was used to determine statistical differences between data sets for pharmacokinetic parameters.

Differences between dissolution profiles of APIs in single dose (reference) and combination (test) were assessed using f_1_ and f_2_ difference and similarity factor testing using the equations:^34^

$$\begin{array}{c} f_{1}=\left\{\left[\sum_{t=1}^{n}\left|R_{t}-T_{t}\right|\right]/\left[\sum_{t=1}^{n}R_{t}\right]\right\}*100\\ f_{2}=50*\log\left\{{\left[1+(1/n)\sum_{t=1}^{n}{\left(R_{t}-T_{t}\right)}^{2}\right]}^{-0.5}*100\right\} \end{array}$$

where *R_t_* and *T_t_* are the percent drug-dissolved value at each time point for the reference and test product, respectively, and *n* is the number of time points.

## Results and discussion

### ODT development

A 500 mg ODT formulation that was both mechanically robust and rapidly disintegrating was developed, which could be produced easily by direct compression to form 13 mm round, flat-faced tablets. The list of excipients was kept low to isolate, as best as possible, the effect of API combination. The formulation consisted of API alongside SSF as a water-soluble lubricant, MCC as a binder and disintegrant, and Pearlitol as a rapidly disintegrating diluent. Compaction forces were applied at a range of 1–2 T, and the effect on ODT properties is shown in Table 2. Hardness values were acceptable from a compaction force of 1.2 T and above. Friability values at all compaction forces were high (>1%), with tablets compressed at and below 1.2 T not withstanding friability testing. Disintegration times at all compaction forces were within 30 s, as advised by the FDA for ODTs^58^ with no significant effect (*P*>0.05) on disintegration with changes in compaction force.

Different concentrations of SSF or Mg stearate (MS) as lubricant were assessed for their effect on ODT properties (Table 3). No significant difference in tablet hardness was demonstrated when SSF concentration was altered. SSF ODT’s displayed greater hardness values than MS, with the exception of SSF at 1% w/w that was not deemed significant. Increasing SSF to 1.5% w/w ensured improved lubricant ability while maintaining high hardness and a low disintegration time. Inclusion of MS at 1% w/w slowed disintegration when compared to all other ODTs, above the 30 s requirement (*P*<0.01).

To combat high friability (>1%), MCC was included as a binder.^59^ Addition of MCC up to 15% w/w (Table 4) improved hardness (*P*<0.01) compared to other concentrations while lowering friability and maintaining rapid disintegration, aided by MCC’s ability to promote water penetration through capillary action due to its high intraparticulate porosity.^60^,^61^ Raising compaction force to 2.2 T lowered friability <1% (0.74%), maintained a low disintegration time of 22.67±2.52 s, and raised hardness to 137.63±2.91 N (data not shown).

The successful formulation was implemented for amlodipine and atorvastatin single dose and FDC ODTs. Formulation compositions for all amlodipine and atorvastatin ODTs are shown in Table 5 and characterization in Table 6.

### HPLC protocol validation

Linearity test solutions were prepared from stocks at six concentrations ranging from 25 to 0.8 μg/mL. Validation of protocols by intraday studies for amlodipine, atorvastatin, and amlodipine/atorvastatin combination (Tables 7–9) shows the methods to be accurate and precise. Method accuracy is demonstrated by multilevel recovery, ranging from 25 to 1.5625 μg/mL. Accurate recovery was exhibited in all instances, ranging from 98.58% to 102.46%. Relative standard deviation (RSD) values representing intraday precision for amlodipine, atorvastatin, and amlodipine/atorvastatin ranged from 1.05% to 7.36%. Instrument precision, tested for by six consecutive injections of the same sample (25 μg/mL), was high, with RSD values ranging from 0.01% to 0.04%. LOQ and LOD values for amlodipine and atorvastatin alone were below 0.6 and 0.2 μg/mL, respectively. LOQ and LOD values for amlodipine/atorvastatin combination were lower still, falling below 0.2 and 0.1 μg/mL, correspondingly.

### Dissolution

**Figure 1:**
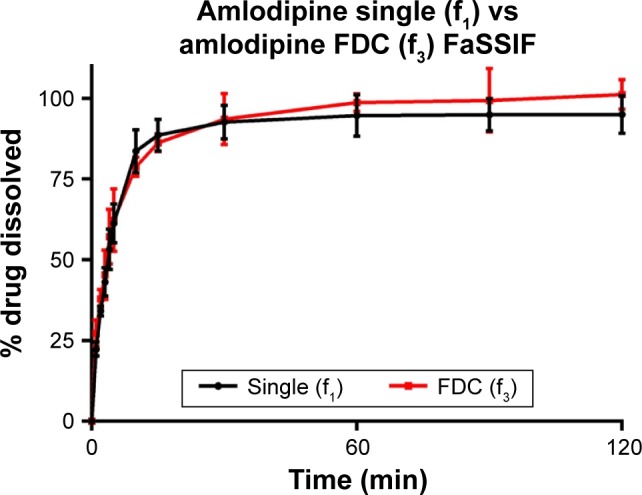
Amlodipine (5 mg) dissolution profiles of single and FDC formulations in fasted-state biorelevant media (900 mL, 37°C) from 500 mg ODTs. Dissolution performed using USP 2 paddle apparatus (mean ± SD, n=3). **Abbreviations:** FDC, fixed-dose combination; ODTs, orally disintegrating tablets; SD, standard deviation; FaSSIF, fasted-state simulated intestinal fluid; USP, United States Pharmacopeia.

**Figure 2:**
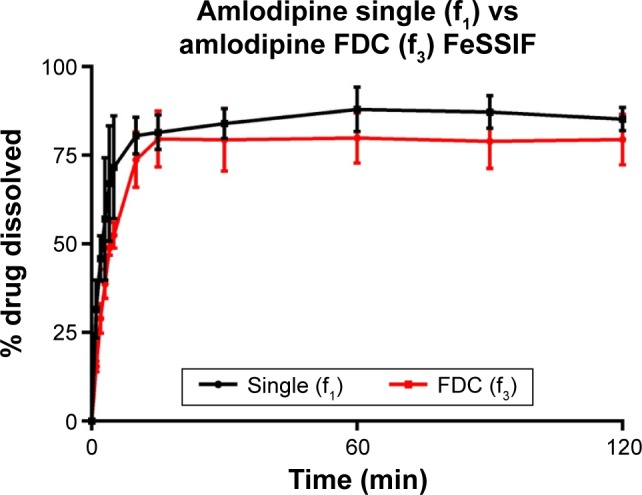
Amlodipine (5 mg) dissolution profiles of single and FDC formulations in fed-state biorelevant media (900 mL, 37°C) from 500 mg ODTs. Dissolution performed using USP 2 paddle apparatus (mean ± SD, n=3). **Abbreviations:** FDC, fixed-dose combination; ODTs, orally disintegrating tablets; SD, standard deviation; FeSSIF, fed-state simulated intestinal fluid.

**Figure 3:**
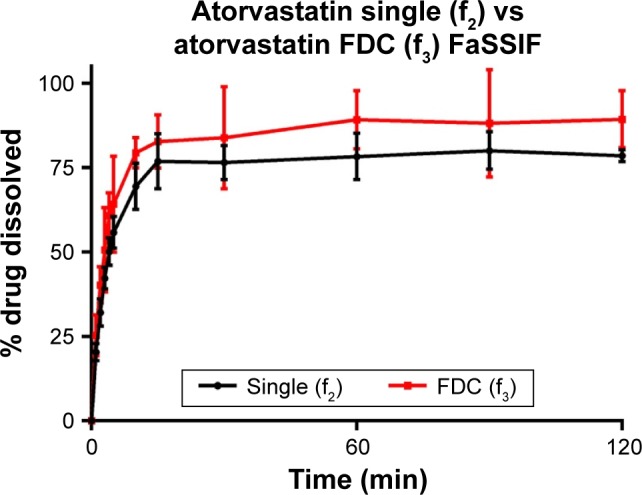
Atorvastatin (10 mg) dissolution profiles of single and FDC formulations in fasted-state biorelevant media (900 mL, 37°C) from 500 mg ODTs. Dissolution performed using USP 2 paddle apparatus (mean ± SD, n=3). **Abbreviations:** FDC, fixed-dose combination; ODTs, orally disintegrating tablets; SD, standard deviation; FaSSIF, fasted-state simulated intestinal fluid.

**Figure 4:**
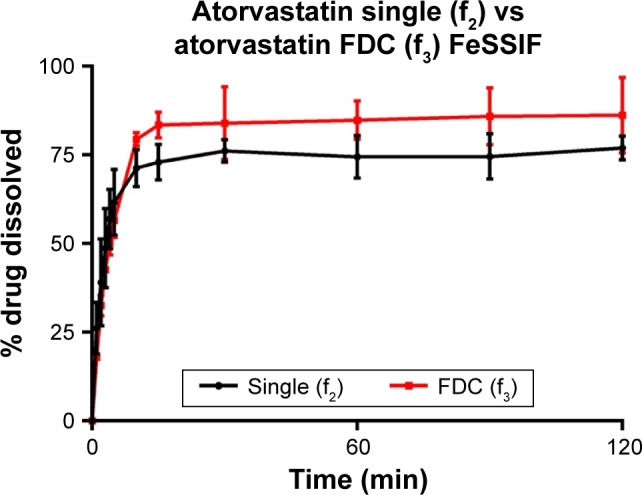
Atorvastatin (10 mg) dissolution profiles of single and FDC formulations in fed-state biorelevant media (900 mL, 37°C) from 500 mg ODTs. Dissolution performed using USP 2 paddle apparatus (mean ± SD, n=3). **Abbreviations:** FDC, fixed-dose combination; ODTs, orally disintegrating tablets; SD, standard deviation; FeSSIF, fed-state simulated intestinal fluid.

Dissolution of API from formulations f_1_–f_3_ was tested in biorelevant media (Figures 1–4). Amlodipine dissolution from f_1_ and f_3_ in FaSSIF was rapid, with >50% dissolution within 5 min. Near-complete dissolution (94.9%) and complete dissolution at (101.2%) were observed in f_1_ and f_3_, respectively. Amlodipine dissolution from f_1_ and f_3_ in FeSSIF peaked at 87.9% and 79.9%, respectively. Difference and similarity testing comparing dissolution profiles of amlodipine from single and combination formulations are shown in Table 10. Difference and similarity testing were used as a tool to compare dissolution profiles in order to predict bioequivalence. In fasted-state media, dissolution of amlodipine from both single and FDC exceeded 85% within 15 min, while f_1_ and f_2_ testing showed no difference between dissolution profiles. Dissolution in FeSSIF did not exceed 85% within 15 min from either single or FDC, with dissolution profiles shown to be different based on f_1_ and f_2_ factors.

Atorvastatin dissolution in FaSSIF was initially rapid, although peaking at 80.0% and 89.3% for single and FDC, respectively. Dissolution profiles in FeSSIF were similar to FaSSIF, with dissolution peaking at 76.9% from single and 86.2% from combination formulations. Greater atorvastatin dissolution from FDCs was not recognized by f_1_ and f_2_ testing (Table 10), with no difference observed between dissolution profiles for single and combination formulations.

Based on difference and similarity testing, only amlodipine in FeSSIF failed to show similar bioequivalence, although >85% dissolution was only observed once. This would suggest that a FDC ODT would likely display similar performance in vivo to a single dose, although based upon current guidelines, this is not assumed for BCS class II compounds. Furthermore, through development of this simple formulation to consistently deliver greater than 85% dissolution (for class I amlodipine), it may be possible to achieve biowaiver status.

### Permeability studies

**Figure 5:**
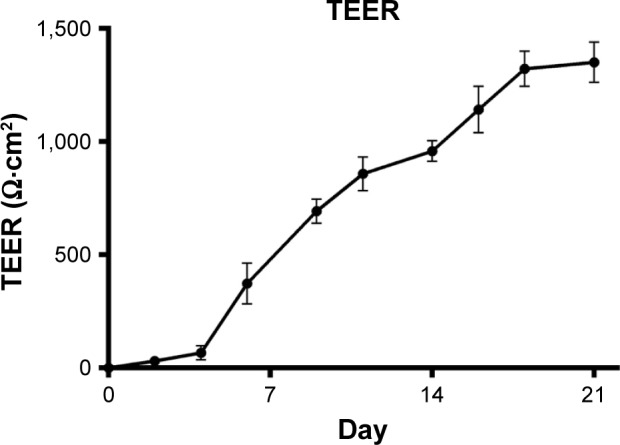
TEER values for Caco-2 monolayers grown on 12 mm Transwell inserts from days 0 to 21 post-seeding. Cells were seeded at a density of 8×10^4^ cells/cm^2^ and maintained in DMEM at 37°C and 5% CO_2_ (mean ± SD, n=6). **Abbreviations:** TEER, transepithelial electrical resistance; DMEM, Dulbecco’s Modified Eagle’s Medium; SD, standard deviation.

**Figure 6:**
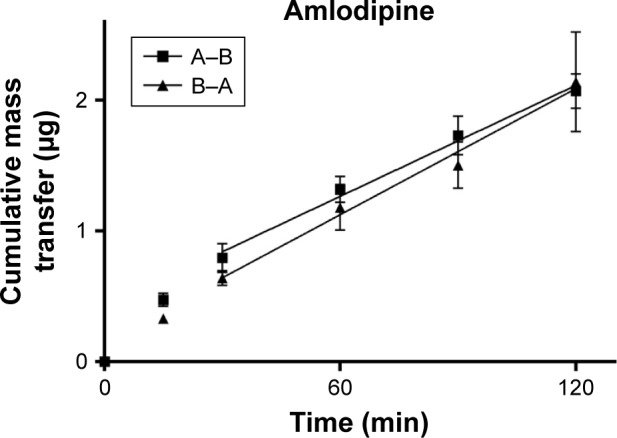
Cumulative mass transfer of amlodipine alone (20 μg/mL) across Caco-2 monolayers (pH 7.4) simulating f_1_. P_app_ values were calculated using the gradient of the linear portion of the curve (mean ± SD, n=3). **Abbreviation:** SD, standard deviation.

**Figure 7:**
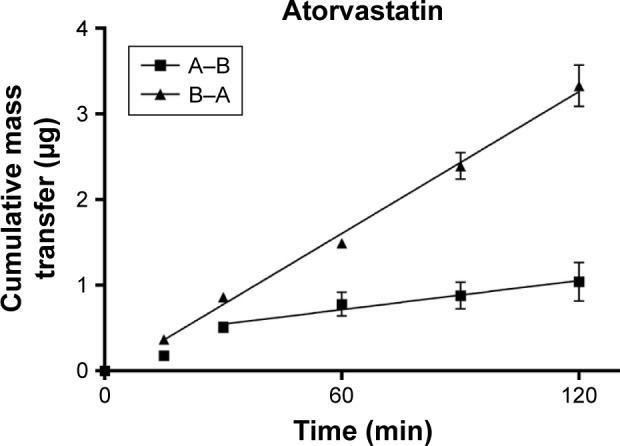
Cumulative mass transfer of atorvastatin alone (40 μg/mL) across Caco-2 monolayers (pH 7.4) simulating f_2_. P_app_ values were calculated using the gradient of the linear portion of the curve (mean ± SD, n=3). **Abbreviation:** SD, standard deviation.

**Figure 8:**
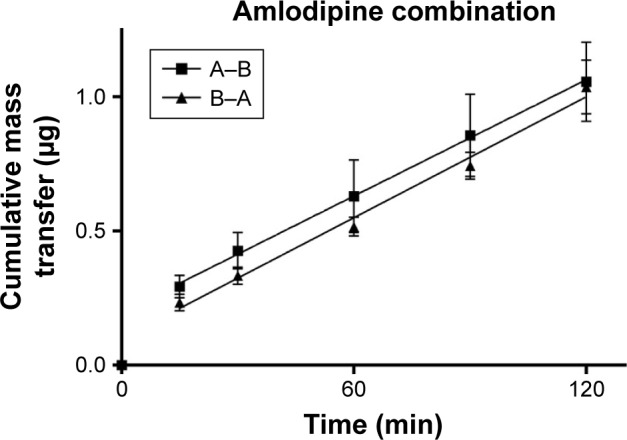
Cumulative mass transfer of amlodipine (20 μg/mL) while in combination with atorvastatin across Caco-2 monolayers (pH 7.4) simulating f_3_. P_app_ values were calculated using the gradient of the linear portion of the curve (mean ± SD, n=3). **Abbreviation:** SD, standard deviation.

**Figure 9:**
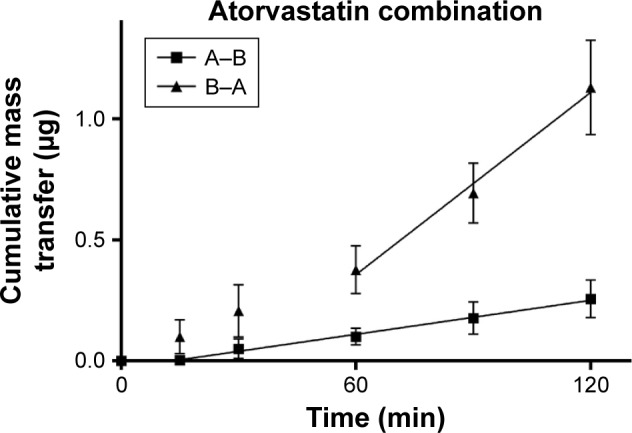
Cumulative mass transfer of atorvastatin (40 μg/mL) while in combination with amlodipine across Caco-2 monolayers (pH 7.4) simulating f_3_. P_app_ values were calculated using the gradient of the linear portion of the curve (mean ± SD, n=3). **Abbreviation:** SD, standard deviation.

TEER values for Caco-2 cells over 21 days are shown in Figure 5, with cell resistance plateauing from day 18 to 1,351.1±88.6 Ω⋅cm^2^ at day 21. Amlodipine and atorvastatin transport across Caco-2 monolayers alone and in combination was measured in both A–B and B–A directions. Drug transport from A to B is shown for amlodipine (Figure 6), atorvastatin (Figure 7), and amlodipine and atorvastatin combination (Figures 8 and 9). The gradient of the linear portion of the curve was used to calculate P_app_ values, summarized in Table 11.

P_app_ values for amlodipine closely mimic those observed by Rausl et al^62^ from both A–B and B–A. Atorvastatin P_app_ values and efflux ratios are similar to those reported by Wu et al.^63^ An efflux ratio of 1.14 for amlodipine indicates passive diffusion of the compound across Caco-2 monolayers, whereas an efflux ratio of 5.02 for atorvastatin suggests active efflux of the API in the B–A direction. Atorvastatin efflux, mediated primarily by P-glycoprotein, has been described previously in the Caco-2 model^63^,^64^ and other cell lines.^65^

When combined with atorvastatin, P_app_ values for amlodipine decreased significantly from A–B (*P*<0.001) and B–A (*P*<0.05), although the efflux ratio remained largely unchanged at 0.96. A decrease in atorvastatin P_app_ value when in combination with amlodipine from A–B was not significant (*P*>0.05) but was significant in the B–A direction (*P*<0.001), with the efflux ratio again maintained at a similar level.

### Clinical trials simulation

The initial simulation of the kinetics of amlodipine and atorvastatin (derived from data presented in Table 1) was used to optimize the absorption P_eff_ and V_ss_ from clinical data sets 1 and 2 for each API. Optimized P_eff_ and V_ss_ were estimated as 1.35×10^−4^ cm/s and 6.12×10^−4^ cm/s for amlodipine and 13.78 and 4.78 l/kg for atorvastatin, respectively. Furthermore, a RAF_P-gp_ of 8.7 was estimated to account for atorvastatin efflux (P-glycoprotein)^63^,^66^ contribution within the small intestine.

**Figure 10:**
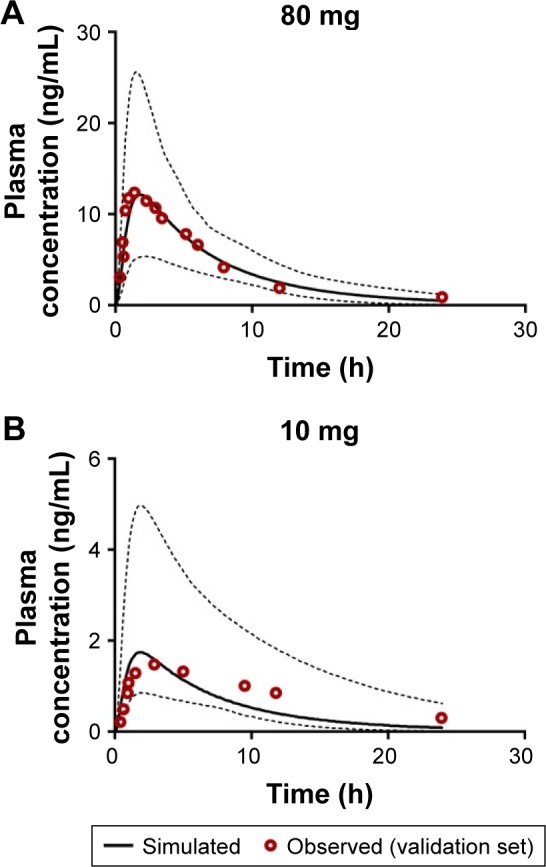
Simulated mean plasma profile after a (**A**) 80 mg and (**B**) 10 mg oral dose of atorvastatin (solid black line). The corresponding observed data points are shown by red open circles. The gray lines represent the 5th and 95th percentiles for the predicted values. All simulations were performed using the minimal PBPK model. **Abbreviation:** PBPK, physiologically based pharmacokinetic.

**Figure 11:**
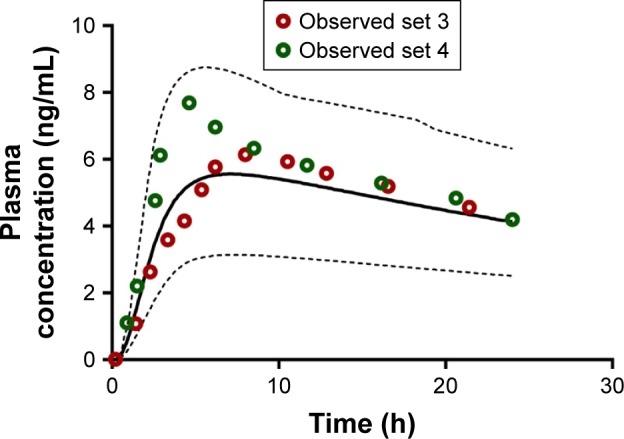
Simulated mean plasma profile after a 10 mg oral dose of amlodipine (solid black line). The corresponding observed data points are shown by red (set 3) or green (set 4) open circles. The gray lines represent the 5th and 95th percentiles for the predicted values. All simulations were performed using the minimal PBPK model. **Abbreviation:** PBPK, physiologically based pharmacokinetic.

Subsequent validation of amlodipine and atorvastatin using validation data sets 3 and 4 for each API was successful and generally centered around the mean simulated profiles and was within the 5th and 95th percentiles of the simulated profiles (Figures 10 and 11).

**Figure 12:**
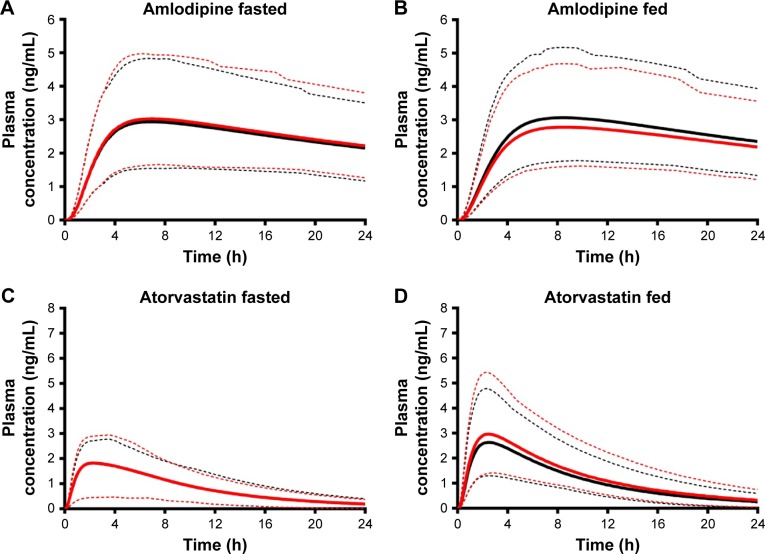
Simulated mean plasma profile after a 5 mg oral dose of amlodipine (**A** and **B**) and 10 mg oral dose of atorvastatin (**C** and **D**) under fasted and fed conditions. Single API formulations are indicated in black and fixed-dose combination in red. Solid lines represent trial mean, and dashed lines represent the 5th and 95th percentiles for the predicted values. All simulations were performed using the minimal PBPK model. **Abbreviations:** API, active pharmaceutical ingredient; PBPK, physiologically based pharmacokinetic.

Simulations to predict the in vivo performance of ODTs in healthy volunteers were used to compare the bioavailability between single and FDC formulations under fasted and fed conditions using dissolution data determined in section “Dissolution”. For amlodipine, the formulation state (single or combined) or dosing state (fasted or fed) had no statistically significant impact on pharmacokinetics (Figure 12A and B). Amlodipine plasma concentrations reached a geometric mean C_max_ of 2.4–2.93 ng/mL in all conditions, yielding a median AUC in the range 53–60 ng/mL⋅h (Table 12).

Fed-state subjects exhibited a longer median t_max_ from 7.12 to 8.12 h in single dose and 7.45 to 8.46 h in combination dose profiles (Table 12). This increased t_max_ in fed subjects is likely a result of delayed gastric emptying and subsequent release of drug into the duodenum^67^ and has been reported previously for amlodipine.^68^

Regarding small intestine, predicted mean fraction dose absorbed (fa) for amlodipine correlated with dissolution profiles, showing significantly different (*P*<0.0001) values between single and combination formulations, 0.92±0.05 and 0.95±0.04 (fasted) and 0.91±0.04 and 0.85±0.05 (fed), respectively.

Atorvastatin plasma profiles similarly showed no statistically significant difference (*P*>0.05) in pharmacokinetic parameters between single and combination doses in fasted subjects (Figure 12C). Atorvastatin plasma concentration increased rapidly after dosing, with a median t_max_ of 2.25 h in fasted and 2.56 h in fed states (Figure 12D) with a similar geometric mean C_max_ of 1.6–1.7 ng/mL and similar AUC (~16–17 ng/mL⋅h) (Table 13) for fasted states. However, under fed conditions there was a significant (*P*<0.05) increase in C_max_ for both single (2.66 ng/mL) and combination (2.96 ng/mL) doses, with an associated increase in the AUC (*P*<0.0001).

**Figure 13:**
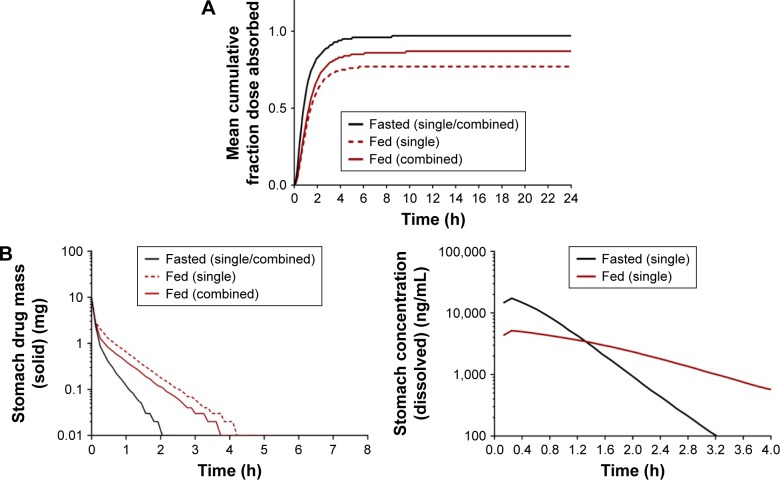

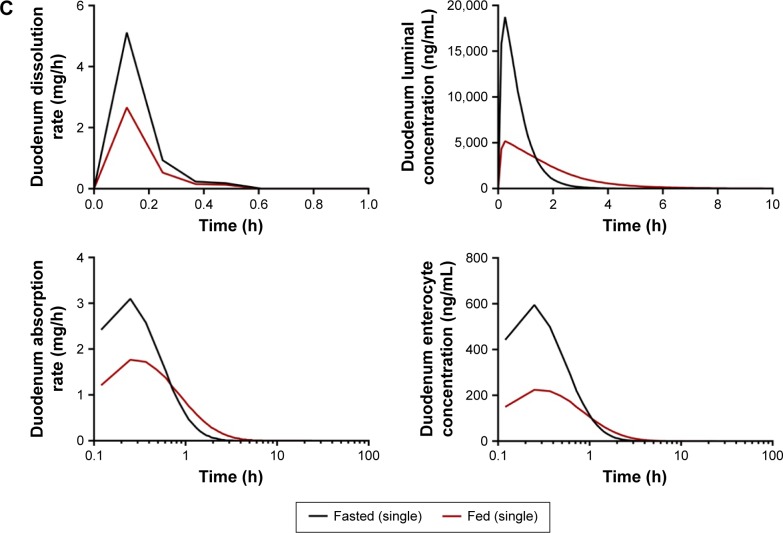
(**A**) Mean cumulative fraction dose absorbed; (**B**) mean solid drug mass in the stomach (left panel) and mean dissolved stomach drug concentration (right panel); (**C**) duodenal dissolution rate (upper left panel), duodenal luminal concentration (upper right panel), duodenal absorption rate (lower left panel), and duodenal enterocyte concentration (lower right panel). Black solid line represents fasted (single/combined), red solid line represents fed (single), and red dashed line represents fed (combined) formulations.

Identical mean fa between single and combination formulations was seen for atorvastatin under fasted state (0.91±0.07). However, under fed conditions, fa was lower (*P*<0.0001) for single compared to combination formulations, at 0.81±0.11 and 0.91±0.09, respectively. It may be prudent to assume that the enhanced AUC and C_max_ for atorvastatin may be due to a positive food effect, given its BCS class II status and, therefore, lipophilic nature.^69^,^70^ However, the impact of fasted/fed status on the fa identified that the absorption across the gut lumen is delayed for both single and combination formulations (Figure 13A). As the cumulative fa is a reflection of events along the entire small-intestine lumen, the impact of food may delay the absorption of atorvastatin into the intestinal enterocytes. However, when considering the mass of dosed atorvastatin within the stomach (10 mg) (Figure 13B), significantly greater quantities of atorvastatin remain undissolved under fed conditions for a longer period of time.

When considered in the context of dissolution and taking the duodenum as an exemplar, the estimated dissolution rates within the duodenum under fasted states are significantly faster than that under fed state, which results in a significantly larger duodenal luminal C_max_ (17,972 ng/mL) compared to the fed state (5,002 ng/mL) (Figure 13C, upper panels). This suggests that the differences between fasted and fed plasma concentrations are a result of changes in the dissolution process of the solid dosage form, otherwise uncaptured when considering the f_1_ and f_2_ tests, due to the dynamic and mechanistic nature of the ADAM-PBPK model.

**Figure 14:**
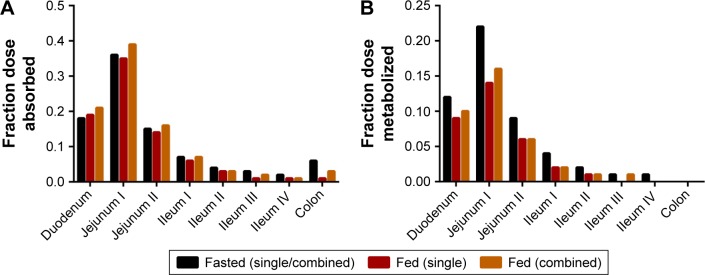
Ab oral regional distribution of (**A**) median fraction dose absorbed and (**B**) median fraction dose metabolized for atorvastatin.

As a result of this reduced dissolution under fed states, the absorption rate of atorvastatin in the duodenum is higher under fasted states with a maximal rate of 3.05 mg/h compared to 1.77 mg/h under fed states, both at 0.28 h (Figure 13C, lower left panel). A consequence of this is a lower overall atorvastatin concentration within the enterocytes and potentially reduced gut metabolic clearance ab orally (Figure 13C, lower right panel). While the fa is relatively invariable ab orally under fasted or fed conditions (Figure 14A), simulations confirmed a noticeable decrease in the fraction of drug metabolized within the enterocytes under all fed conditions (Figure 14B). Atorvastatin possesses a low oral bioavailability (F<10%) and this is primarily a function of its high first-pass metabolism. Under fed conditions, this decrease in regional ab oral fraction of dose metabolized would result in an increased overall oral bioavailability (F_oral_ = f_a_ × f_g_ × f_h_) and is, therefore, the primary cause of the increased C_max_ observed under fed conditions for both single and combined formulations (Figure 12D).

When considering the physical process of drug absorption, it is important to conceptualize the small intestine and associated distribution of metabolic enzymes ab orally. With this in mind, CYP3A4 expression would be greatest duodenally and decrease longitudinally ab orally.^71^,^72^ As a result of this, the delayed absorption of drug across the gut wall (as a result of reduced dissolution) under fed states would result in a longer residency of solid (undissolved) drug in the proximal small intestine lumen, which would be susceptible to transit along the gut lumen until dissolution was complete, resulting in absorption of atorvastatin more distally.

Atorvastatin is a BCS class II compound where solubility/dissolution is the rate limiting step for absorption, coupled with often high metabolism. The oral bioavailability of atorvastatin is relatively low, indicating significant metabolic clearance.^73^ Fed state often results in slower gastric emptying and the presence of food alters luminal composition through an increase in bile salts. Indeed, post-prandial changes can often contribute to an increased bioavailability of many class II compounds. A review by Gu et al^69^ compared food effects on 92 sets of clinical data and demonstrated that 71% of BCS class II compounds resulted in an enhancement of bioavailability following meals.

Although dissolution studies in FaSSIF and FeSSIF are useful, the mechanistic nature of the ADAM model, coupled with a detailed ab oral consideration of geometric, physiological and biochemical variations, allows a greater understanding of the role of small intestine physiology on the process of oral drug absorption – an understanding that would otherwise not be captured by in vitro dissolution studies or subsequent statistical analysis (ie, f_1_ and f_2_ testing). Data for dissolution, permeability and simulated clinical trials can be accessed online.

## Conclusion

An ODT formulation was developed and characterized, demonstrating acceptable performance for hardness, friability, and disintegration time and was subsequently used for formulation of low-dose ODTs for amlodipine and atorvastatin, alone and in FDC. Clinical trial simulations using an ADAM-PBPK model were able to predict the in vivo pharmacokinetics of amlodipine and atorvastatin for comparison of the performance of FDCs against single-dose formulations. In vitro dissolution data were incorporated to more accurately model the performance of the developed formulation and P_app_ values to model intestinal absorption.

Dissolution profiles showed no differences based on f_1_ and f_2_ testing between FDC and single-dose formulations, with the exception of amlodipine in FeSSIF. All FDC formulations were shown to be bioequivalent based on clinical trial simulations in fasted and fed subjects (AUC, C_max_, and t_max_), despite the failure of amlodipine in FeSSIF based on f_1_ and f_2_, adding incentive for the use of in silico simulation. Furthermore, the demonstration of bioequivalence through f_1_ and f_2_ and PBPK simulation for atorvastatin, a class II compound, adds weight to the argument for the applicability of class II inclusion in biowaiver applications, ideally in combination with PBPK modeling. Atorvastatin enjoyed a greater C_max_ and AUC in the fed state, due to an extended transit along the gut lumen as a result of poor dissolution. The attenuating expression of CYP3A4 distally along the gut meant that less atorvastatin was thus metabolized in the fed state. This food effect on the pharmacokinetic parameters for atorvastatin was not evident from in vitro investigation alone, further demonstrating the power and applicability of mechanistic PBPK modeling.

## Figures and Tables

**Table 1:** Input parameter values and predicted PBPK values for simulation of pharmacokinetics of amlodipine and atorvastatin

Parameters	Amlodipine	Atorvastatin
Type	Diprotic base	Monoprotic acid
MW	408.88	588.2
LogP	3.43^74^	5.7
pKa	9.4, 1.90^74^	4.46
fu	0.07^75^	0.051
V_ss_ (L/kg)^a^	Predicted PBPK/PE	Predicted PBPK/PE
B:P ratio	1	0.61
CLpo (L/min)	24.8	949
CLint_3A4_^b^	–	8.28
P_eff_ (cm/s)	PE	PE
Jmax_P-gp_ (pmoL/cm^2^/min)	–	151^66^
Km_P-gp_ (μM)	–	115^66^
RAF_P-gp_	–	PE

**Notes:**

a V_ss_ was determined from calculation of tissue partitions coefficients within Simcyp or parameter estimated.

b In vitro intrinsic metabolic clearance (CLint) was calculated using Simcyp Retrograde Calculator from in vivo oral clearance and assuming f_a_ =1, f_g_=0.24^76^ with CYP3A4 being the predominant metabolic pathway.^77^

**Abbreviations:** MW, molecular weight; fu, plasma unbound fraction; V_ss_, steady-state volume of distribution; B:P ratio, blood-to-plasma ratio; P_eff_, human effective permeability; PBPK, physiologically based pharmacokinetic; PE, parameter estimation; RAF, relative activity factor; CLpo, oral clearance.

**Table 2:** ODTs consisting of SSF (0.5% w/w) and Pearlitol Flash as a diluent

Compaction force (T)	Hardness (N)	Disintegration time (s)	Friability (%)
1	51.40±0.26	19.33±1.53	–
1.2	68.27±5.56	20.67±4.16	–
1.4	78.23±2.96	18.33±2.52	3.97
1.6	99.37±5.28	21.33±0.58	2.46
1.8	99.83±13.67	19.67±1.15	2.29
2	100.17±7.97	20.33±0.58	1.97

**Notes:** The effect of altering compaction force on tablet properties is shown. Data presented as mean ± standard deviation.

**Abbreviations:** ODTs, orally disintegrating tablets; SSF, sodium stearyl fumarate.

**Table 3:** ODTs containing either MS or SSF as a lubricant

Lubricant	Hardness (N)	Disintegration time (s)	Friability (%)
SSF 0.5% w/w	100.17±7.97	20.33±0.58	1.97
SSF 1% w/w	96.27±6.87	18.67±1.15	1.62
SSF 1.5% w/w	101.03±2.35	21.67±0.58	1.71
MS 0.5% w/w	82.07±7.72	25.33±2.52	1.61
MS 1% w/w	61.90±2.55	43.67±9.71	2.83

**Notes:** The effect of changing lubricant and lubricant concentration on ODT properties is shown. Data presented as mean ± standard deviation.

**Abbreviations:** ODTs, orally disintegrating tablets; MS, Mg stearate; SSF, sodium stearyl fumarate.

**Table 4:** Inclusion of MCC as a binder in ODTs comprising SSF (1.5% w/w) and Pearlitol as a diluent

MCC	Hardness (N)	Disintegration Time (s)	Friability (%)
5% MCC w/w	102.03±1.62	19.33±1.15	1.67
10% MCC w/w	106.00±3.68	20.67±1.15	1.48
15% MCC w/w	119.50±3.90	20.33±1.15	1.04

**Notes:** MCC concentrations are in % w/w. Data presented as mean ± standard deviation.

**Abbreviations:** MCC, micro-crystalline cellulose; ODTs, orally disintegrating tablets; SSF, sodium stearyl fumarate.

**Table 5:** ODT formulations for individual dose and FDC ODTs

API/Excipient	Amlodipine (1%)	Atorvastatin (2%)	Amlodipine + Atorvastatin (1%+2%)
	f_1_	f_2_	f_3_
Amlodipine besylate	6.95		6.95
Atorvastatin calcium		10.85	10.85
Pearlitol Flash	410.55	406.65	399.7
SSF (1.5%)	7.5	7.5	7.5
MCC (15%)	75	75	75

**Notes:** Values for APIs and excipients are given in % w/w for 500 mg tablets. All formulations underwent compaction at 2.2 T with a 6 s dwell time.

**Abbreviations:** ODT, orally disintegrating tablet; FDC, fixed-dose combination; SSF, sodium stearyl fumarate; MCC, micro-crystalline cellulose; APIs, active pharmaceutical ingredients.

**Table 6:** Individual and FDC ODT properties

f	Hardness (N)	Porosity	Disintegration time (s)	Friability (%)
f_1_	108.00±8.35	0.23±0.15	25.33±3.21	0.71
f_2_	114.40±4.10	0.25±0.00	24.00±3.00	1.02
f_3_	117.77±8.97	0.24±0.02	21.67±1.53	0.73

**Notes:** All formulations underwent compaction at 2.2 T with a 6 s dwell time. Data presented as mean ± standard deviation.

**Abbreviations:** FDC, fixed-dose combination; ODT, orally disintegrating tablet.

**Table 7:** HPLC method validation for detection of amlodipine

Actual conc of amlodipine (µg/mL)	Calculated conc of amlodipine (µg/mL)	RSD (%)	Recovery (%)
25	25.02±1.30	5.19	100.10±5.20
12.5	12.49±0.57	4.59	99.95±4.58
6.25	6.16±0.29	4.69	98.58±4.62
3.125	3.11±0.13	4.13	99.54±4.12
1.5625	1.59±0.06	3.68	101.83±3.75
Instrument precision (% RSD) =0.02			
Mean % recovery =100.00±1.18			
RSD % recovery =0.01			
LOD =0.17 μg/mL			
LOQ =0.57 μg/mL			
Correlation coefficient = 0.99997			

**Notes:** Data for linearity (correlation coefficient), instrument precision, accuracy (recovery), precision (% RSD), LOD, and LOQ are displayed. Data presented as mean ± standard deviation.

**Abbreviations:** HPLC, high performance liquid chromatography; RSD, relative standard deviation; LOD, limit of detection; LOQ, limit of quantification.

**Table 8:** HPLC method validation for detection of atorvastatin

Actual conc of atorvastatin (µg/mL)	Calculated conc of atorvastatin (µg/mL)	RSD (%)	Recovery (%)
25	25.05±1.44	5.76	100.19±5.77
12.5	12.42±0.66	5.34	99.34±5.31
6.25	6.23±0.38	6.08	99.72±6.07
3.125	3.08±0.23	7.36	98.42±7.24
1.5625	1.60±0.10	6.25	102.46±6.40
Instrument precision (% RSD) =0.04			
Mean % recovery =100.02±1.51			
RSD % recovery =0.02			
LOD =0.12 μg/mL			
LOQ =0.40 μg/mL			
Correlation coefficient =0.99996			

**Notes:** Data for linearity (correlation coefficient), instrument precision, accuracy (recovery), precision (% RSD), LOD, and LOQ are displayed. Data presented as mean ± standard deviation.

**Abbreviations:** HPLC, high performance liquid chromatography; RSD, relative standard deviation; LOD, limit of detection; LOQ, limit of quantification.

**Table 9:** HPLC validation for simultaneous detection of amlodipine and atorvastatin

Actual conc (µg/mL)	Calculated conc (µg/mL)	RSD (%)	Recovery (%)
Amlodipine			
25	25.04±1.16	4.65	100.15±4.65
12.5	12.43±0.64	5.10	99.46±5.10
6.25	6.22±0.35	5.64	99.56±5.64
3.125	3.12±0.18	5.85	99.89±5.85
1.5625	1.58±0.09	6.06	100.96±6.06
Instrument precision (% RSD) =0.03			
Mean % recovery =100.01±0.60			
RSD % recovery =0.01			
LOD =0.04 μg/mL			
LOQ =0.13 μg/mL			
Correlation coefficient =0.99998			
Atorvastatin			
25	25.01±0.26	1.05	100.03±1.05
12.5	12.50±0.16	1.28	99.97±1.28
6.25	6.23±0.14	2.17	99.72±2.17
3.125	3.11±0.08	2.42	99.64±2.42
1.5625	1.56±0.04	2.68	100.03±2.68
Instrument precision (% RSD) =0.02			
Mean % recovery =99.88±0.18			
RSD % recovery =0.00			
LOD =0.05 μg/mL			
LOQ =0.17 μg/mL			
Correlation coefficient =1			

**Notes:** Data for linearity (correlation coefficient), instrument precision, accuracy (recovery), precision (% RSD), LOD, and LOQ are displayed. Data presented as mean ± standard deviation.

**Abbreviations:** HPLC, high performance liquid chromatography; RSD, relative standard deviation; LOD, limit of detection; LOQ, limit of quantification.

**Table 10:** Comparison of dissolution profiles for each compound from single and FDC formulations in FaSSIF and FeSSIF media, by difference factor f_1_ and similarity factor f_2_ testing

Compounds	>85% dissolution ≤15 min	f_1_	f_2_	Results
Amlodipine				
FaSSIF	Yes	5.08	70.80	Pass
FeSSIF	No	15.92	45.40	Fail
Atorvastatin				
FaSSIF	No	14.16	53.81	Pass
FeSSIF	No	13.24	54.59	Pass

**Notes:** Dissolution profiles are considered similar if the f_1_ value is below 15, and the f_2_ value is above 50.

**Abbreviations:** FDC, fixed-dose combination; FaSSIF, fasted-state simulated intestinal fluid; FeSSIF, fed-state simulated intestinal fluid.

**Table 11:** P_app_ vales for amlodipine and atorvastatin alone and in combination in A–B and B–A directions across Caco-2 monolayers at pH 7.4 in both compartments (n=3)

Compounds	P_app_ (10^−6^ cm s^−1^)		Efflux ratio
	A–B	B–A	
Amlodipine	8.34±0.32	9.51±1.70	1.14
Atorvastatin	2.03±0.96	10.18±0.71	5.02
Amlodipine combination	5.40±0.48	5.18±0.29	0.96
Atorvastatin combination	0.87±0.18	4.59±0.44	5.29

**Note:** Data presented as mean ± standard deviation.

**Abbreviations:** A–B, apical to basolateral; B–A, basolateral to apical.

**Table 12:** Summary of pharmacokinetic parameters for amlodipine (5 mg) under fasted and fed conditions

Parameters	Amlodipine fasted		Amlodipine fed	
	Single	Combined	Single	Combined
AUC (ng/ml⋅h)	53.42 (32.12–75.69)	55.12 (30.12–74.11)	60.11 (42.75–81.94)	55.36 (35.69–78.91)
C_max_ (ng/ml)	2.45 (1.15)	2.57 (1.23)	2.87 (1.67)	2.89 (1.17)
t_max_ (h)	7.12 (5.92–8.21)	7.45 (5.21–9.72)	8.12 (6.96–9.54)	8.46 (7.95–9.87)

**Note:** Geometric mean (SD) reported for C_max_ and median (range) for AUC and t_max_.

**Abbreviation:** SD, standard deviation.

**Table 13:** Summary of pharmacokinetic parameters for atorvastatin (10 mg) under fasted and fed conditions

Parameters	Atorvastatin fasted		Atorvastatin fed	
	Single	Combined	Single	Combined
AUC (ng/mL⋅h)	16.24 (2.78–64.45)	17.15 (3.04–62.99)	25.77 (5.47–75.17)	29.46 (6.73–87.72)
C_max_ (ng/mL)	1.61 (1.27)	1.72 (1.31)	2.66 (1.80)	2.96 (1.97)
t_max_ (h)	2.25 (1.51–7.86)	2.28 (1.45–5.31)	2.56 (1.45–5.25)	2.71 (1.45–5.72)

**Note:** Geometric mean (SD) reported for C_max_ and median (range) for AUC and t_max_.

**Abbreviation:** SD, standard deviation.
